# Outcomes of Femtosecond Laser-Assisted Arcuate Keratotomy in the Management of Keratoplasty-Related Astigmatism

**DOI:** 10.3390/jcm14134526

**Published:** 2025-06-26

**Authors:** Majed S. Alkharashi, Mohammed M. Abusayf, Khalid B. Alburayk, Abdulmajeed S. Alkharashi

**Affiliations:** 1 Department of Ophthalmology, College of Medicine, King Saud University, Riyadh 12372, Saudi Arabia; dr.khalidba11@gmail.com (K.B.A.); asalkharashi@gmail.com (A.S.A.); 2 Refractive Surgery and Myopia Research Group, Ophthalmology and Visual Sciences Research Center, King Saud University, Riyadh 12629, Saudi Arabia

**Keywords:** femtosecond, keratoplasty, astigmatism, vision, topography, keratoconus, arcuate, astigmatic, keratotomy

## Abstract

**Background/Objectives**: Post-keratoplasty astigmatism can limit visual recovery even after successful corneal transplantation. Femtosecond laser-assisted arcuate keratotomy (FSAK) has emerged as a method to reduce high residual astigmatism and enhance visual outcomes. This study aimed to evaluate the outcome of FSAK in treating astigmatism following keratoplasty. **Methods**: This retrospective study included 32 eyes from 31 patients who underwent FSAK after keratoplasty. Inclusion required complete suture removal, regular corneal topography, and the absence of additional ocular pathology or prior intraocular surgery. Data collected included uncorrected (UCVA) and best-spectacle-corrected visual acuity (BSCVA), manifest refraction, and tomographic parameters. The primary outcomes were changes in visual, refractive, and tomographic measures across the entire cohort, with further subgroup analysis between penetrating keratoplasty (PKP) and lamellar keratoplasty (LKP) eyes. Secondary outcomes were documentation of complications. **Results**: UCVA improved significantly from 0.92 ± 0.33 to 0.58 ± 0.39 LogMAR (*p* < 0.001). BSCVA showed a non-significant trend toward improvement from 0.32 ± 0.21 to 0.26 ± 0.22 LogMAR (*p* = 0.158). The manifest cylinder reduced significantly from −6.15 ± 2.75 D to −4.49 ± 2.92 D (*p* = 0.037). Corneal topography revealed significant postoperative steepening in keratometric values. While overall outcomes were comparable between the subgroups, LKP eyes demonstrated a greater myopic shift and a higher rate of overcorrection, whereas PKP eyes tended toward undercorrection. **Conclusions**: FSAK appears to be an effective approach for reducing post-keratoplasty astigmatism and improving uncorrected visual acuity. Given the biomechanical differences between graft types, individualized treatment planning based on graft characteristics may enhance surgical predictability and optimize outcomes.

## 1. Introduction

Astigmatic keratotomy refers to incision-based procedures to treat corneal astigmatism and typically include limbal relaxing incisions (LRI), arcuate keratotomies (AK), and transverse keratotomy (TK). Astigmatic keratotomy and arcuate keratotomy (AK) are often used interchangeably. Arcuate keratotomy involves making arc-shaped corneal incisions, typically in the peripheral cornea, to flatten the steep meridian [1,2,3]. Femtosecond laser-assisted arcuate keratotomy (FSAK) enhances precision and safety in these procedures [2,4,5]. Penetrating keratoplasty (PKP) refers to full-thickness corneal transplantation, while lamellar keratoplasty (LK) involves selective replacement of the corneal stroma preserving the endothelium [6,7]. Post-keratoplasty high corneal astigmatism represents a primary concern that could potentially hinder visual recovery and rehabilitation following a corneal transplantation procedure and could lead to optically failed keratoplasty despite excellent anatomical success [8,9]. Astigmatism develops due to changes in the corneal structure, such as scarring, suture tension, or irregularities at the graft–host interface [9]. Astigmatism can be corrected with glasses or contact lenses. In addition, several surgical interventions can be used to treat residual astigmatism in this context, such as AK, laser refractive surgery, and/or phakic intraocular implants [10,11,12]. Excimer laser ablation has shown satisfactory outcomes for up to 6D cylinders and in cases of irregular astigmatism; however, their ability to correct high regular astigmatism above 6D is limited [11,13]. In addition, postoperative haze may further complicate the case, particularly in eyes that have undergone keratoplasty [9,14]. As a result, AK is frequently used as the first treatment for these individuals [1,2,4,5,10,11,12].

Nevertheless, AK, even when assisted by femtosecond laser technology, still faces some challenges. One of the limitations of AK is its inherent unpredictability, particularly in cases with high astigmatism in grafted corneas [3,15,16]. A retrospective study examining 89 cases of post-keratoplasty astigmatism found that approximately 33% of treatment inaccuracies could not be explained by identifiable factors [17]. The remaining inaccuracies were attributed to elements such as the preoperative level of astigmatism, arc length, incision depth, and the diameter of the incisions made during the procedure [17,18]. Furthermore, selecting the appropriate nomogram and making individualized adjustments to the settings for each patient remains a challenge, underscoring the need for personalized approaches in AK treatment [15,17,19].

This study aims to report the outcome of FSAK in post-keratoplasty patients in a tertiary ophthalmic center.

## 2. Materials and Methods

### 2.1. Study Design

This retrospective study included 32 eyes from 31 patients who underwent AK after keratoplasty between January 2017 and January 2024. Data collected for each patient included uncorrected visual acuity (UCVA), best spectacle-corrected visual acuity (BSCVA), ophthalmic examination findings, refraction, and corneal tomographic evaluation using Pentacam (Oculus Systems, Wetzlar, Germany). Inclusion criteria included patients who had previously undergone keratoplasty, either full or partial thickness keratoplasty, and subsequently underwent AK after complete removal of all corneal sutures in otherwise healthy eyes were included in the study. Primarily, patients were selected by surgeons for AK candidacy based on the presence of regular corneal astigmatism, as demonstrated by corneal topography after the complete removal of sutures. Two categories of patients were included: (1) those who showed improvement with best spectacle-corrected visual acuity (BSCVA) and (2) those who exhibited suboptimal BSCVA improvement but demonstrated clear visual improvement with rigid contact lens fitting, indicating the presence of a regular astigmatic component that was undercorrected with spectacles. Exclusion criteria included eyes with concurrent ocular diseases, such as glaucoma or retinal conditions or those with a history of additional ocular surgeries, including retinal surgery, cataract extraction, or surface laser procedures; they were excluded from the analysis. This study was approved by the Institutional Review Board of King Saud University (E-25-9841) and conducted in accordance with the Declaration of Helsinki.

Primary outcomes included changes in UCVA, BSCVA, manifest refraction, and tomographic parameters, analyzed for the overall cohort and stratified by graft type (PKP and LKP). Secondary outcomes focused on the incidence of postoperative complications. Data were compiled, verified, and analyzed using the Statistical Package for Social Sciences (version 26, SPSS Inc., Chicago, IL, USA) version 26. For each attribute and demographic variable, descriptive statistics (i.e., frequencies, percentages, and measures of central tendency and dispersion, where applicable) were generated. The Wilcoxon test and t-test were used to calculate differences between groups. A comparison was made to assess the significance of the outcomes pre- and post-treatment at 1, 3, 6, and 12 months (±5–14 m for all follow-up time points). A *p*-value < 0.05 was considered statistically significant. An improvement of three lines of UCVA according to Early Treatment Diabetic Retinopathy Study (ETDRS) lines was used for successful outcome.

Vector analysis was performed using standard methods to assess the effectiveness and accuracy of astigmatic correction following AK based on the calculation of surgically induced astigmatism (SIA), target-induced astigmatism (TIA), difference vector (DV), correction index (CI), and index of success (IOS).

### 2.2. Surgical Technique

Under topical anesthesia, the geometric center of the graft was marked. Corneal thickness was measured using ultrasonic pachymetry at the 6.5 mm optical zone along the planned incision meridians and cross-verified with corneal tomography. In each case, two anterior arcuate incisions were created along the patient-specific steep meridian using the iFS femtosecond laser (Johnson & Johnson Surgical Vision, Inc., Santa Ana, CA, USA). Arc lengths were individualized based on the degree of refractive astigmatism and typically ranged from 45° to 70°, as determined using a modified arcuate incision nomogram from previously published work. The incisions were made at a depth corresponding to 70–85% of the thinnest measured corneal thickness. Laser parameters were set as follows: 6.5 mm optical zone diameter, 2.50 mJ anterior side-cut energy, 90° side-cut angle, and a 5-spot offset from the anterior side-cut. Immediately after laser application, a Sinskey hook was used to open the incisions.

#### Postoperative Management and Follow-Up

All patients were prescribed a tapering regimen of topical corticosteroids over one month and a course of topical antibiotics for one week. Follow-up visits were scheduled at day 1, week 1, month 1, month 3, month 6, year 1, and annually thereafter, unless further intervention was required.

## 3. Results

### 3.1. Demographics and Clinical Characteristics

A total of 32 eyes from 31 patients were included in the study, with a mean age of 35.5 ± 8.1 years (range 17–55). The majority of the participants were male (61.3%), and keratoconus (KC) was the most common indication for keratoplasty (90.7%). Other diagnoses included macular dystrophy, mixed astigmatism, and post-LASIK ectasia, each contributing 3.1%. Complications observed included corneal keloid (1 case) and infectious keratitis at the AK wound (1 case). PKP was the more frequently performed procedure (57%) compared to LKP (43%).

### 3.2. Visual Acuity and Refractive Outcomes

On the last follow-up, the mean UCVA improved from 0.92 ± 0.33 LogMAR (20/165) preoperatively to 0.58 ± 0.39 LogMAR (20/76) postoperatively (*p* < 0.001). BSCVA improved from 0.32 ± 0.21 LogMAR (20/42) preoperatively to 0.26 ± 0.22 LogMAR (20/36) postoperatively, although this change was not statistically significant (*p* = 0.158). Refractive outcomes demonstrated a reduction in the mean manifest cylinder (M) from −6.15 ± 2.75 D to −4.49 ± 2.92 D (*p* = 0.037). The cylinder axis and manifest sphere exhibited changes, with the latter showing a significant shift from −1.13 ± 3.3 D preoperatively to −2.72 ± 4.05 D postoperatively (*p* = 0.040) (Table 1).

#### 3.2.1. Visual and Refractive Outcomes Based on Transplant Type

##### Penetrating Keratoplasty (PKP)

In the PKP subgroup, UCVA improved significantly from 1.04 ± 0.37 LogMAR (20/220) preoperatively to 0.53 ± 0.42 LogMAR (20/67) postoperatively (*p* = 0.001). However, no significant change was observed in BSCVA. The mean cylinder reduced from −5.88 ± 2.79 D to −4.86 ± 2.33 D (*p* = 0.270).

##### Lamellar Keratoplasty (LKP)

In the LKP subgroup, UCVA improved from 0.78 ± 0.22 LogMAR (20/120) to 0.63 ± 0.35 LogMAR (20/85). BSCVA changes were also not significant. (*p* = 0.662). A reduction in the manifest cylinder from −6.48 ± 2.79 D to −4.06 ± 3.59 D approached significance (*p* = 0.086), and a significant shift in the manifest sphere from −0.98 ± 2.77 D to −4.5 ± 4.02 D was noted (*p* = 0.031).

### 3.3. Tomographic Changes

AK across all keratoplasty cases resulted in a notable reshaping of the corneal surface. The maximum keratometry (Kmax) increased from 48.31 ± 5.75 preoperatively to 51.7 ± 6.44 at the last visit (*p* = 0.001), demonstrating a steepening effect of the procedure. Similarly, the flat keratometry (Flat K) increased significantly from 41.21 ± 3.79 preoperatively to 44.22 ± 2.78 at the last visit (*p* = 0.001), demonstrating corneal steepening in the flat meridian. Steep keratometry (Steep K) showed flattening changes from 50.36 ± 3.03 to 48.72 ± 3.173 (*p* = 0.007). The anterior corneal cylinder decreased significantly from 9.14 ± 3.1 to 4.86 ± 2.22 (*p* < 0.001), reflecting a successful reduction in astigmatism. Posterior corneal astigmatism showed minimal changes, suggesting limited posterior corneal reshaping with this procedure (Table 2).

#### 3.3.1. Tomographic Changes Based on Transplant Type

##### Penetrating Keratoplasty (PKP)

For the PKP subgroup, significant tomographic changes were observed. The Kmax increased from 50.38 ± 6.58 preoperatively to 53.59 ± 6.81 at the last visit (*p* = 0.002), confirming the steepening effects. Flat K increased significantly from 40.98 ± 4.03 to 42.89 ± 2.52 (*p* = 0.007). Steep K showed a significant reduction from 50.16 ± 2.61 preoperatively to 46.85 ± 3.07 at the last visit (*p* = 0.007). The anterior corneal cylinder reduced substantially from 9.16 ± 3.26 to 4.54 ± 2.8 (*p* < 0.001), showcasing effective astigmatism correction. Posterior corneal astigmatism changes were insignificant, highlighting the limited impact on the posterior cornea (*p* > 0.05) (Table 3).

##### Lamellar Keratoplasty (LKP)

The LKP subgroup demonstrated an increase in Kmax from 45.8 ± 3.36 to 49.52 ± 5.43 at the last visit (*p* = 0.001), indicating significant steepening. Flat K increased significantly from 41.59 ± 3.77 to 45.65 ± 2.37 (*p* = 0.005), consistent with corneal reshaping. The anterior corneal cylinder reduced markedly from 9.53 ± 2.76 to 5.22 ± 1.3 (*p* < 0.001). Posterior corneal astigmatism showed minimal changes, with a reduction from 1.35 ± 0.53 preoperatively to 0.92 ± 0.3 at the last visit (*p* = 0.006) (Table 4).

### 3.4. Success and Non-Success Groups

Preoperative characteristics were compared between successful (*n* = 23) and non-successful (*n*= 9) outcomes. No significant differences were noted in age, gender, or preoperative refractive parameters. The mean UCVA and BSCVA did not differ significantly between groups. Additionally, no significant differences were observed in K values, anterior or posterior corneal astigmatism, or axis measurements (Table 5).

### 3.5. Comparative Outcomes Between PKP and LKP

Visual and refractive outcomes, including safety and efficacy indices, were compared between PKP and LKP groups. Both groups demonstrated comparable improvement in refractive parameters, with no statistically significant differences in indices such as surgically induced astigmatism (SIA) magnitude, flattening effect, or torque effect (Table 6).

### 3.6. Vector Analysis of the Astigmatism for PKP Versus LKP

In the comparison between PKP and LKP, no statistically significant differences were observed across all evaluated parameters. The safety index was slightly higher in LKP (1.35 ± 1.2) compared to PKP (0.96 ± 1.08), while the efficacy index also showed a minor increase in LKP (3.00 ± 1.94) over PKP (2.17 ± 2.22), though neither reached statistical significance (*p* = 0.415, *p* = 0.325, respectively). Vector analysis indicated similar target-induced astigmatism (TIA) values between PKP (8.84 ± 3.38 D) and LKP (8.56 ± 2.80 D) (*p* = 0.822). Overcorrection was more frequent in LKP (54.5%) than in PKP (28.6%), while undercorrection was more common in PKP (71.4%) compared to LKP (45.5%), though these differences were not statistically significant (*p* = 0.241). The surgically induced astigmatism (SIA) magnitude was higher in LKP (8.65 ± 5.74 D) than in PKP (7.15 ± 4.59 D) (*p* = 0.456). Mean error (ME) was closer to zero in LKP (−0.15 ± 4.40 D) compared to PKP (−2.26 ± 3.95 D) (*p* = 0.222), and the angle of error (AE) showed more deviation in LKP (−13.84 ± 29.62°) than in PKP (−4.33 ± 12.68°) (*p* = 0.289). The difference vector (DV) remained comparable between PKP (5.65 ± 2.40 D) and LKP (6.00 ± 2.65 D) (*p* = 0.747).

Regarding the flattening/steepening effect, the magnitude was greater in LKP (7.37 ± 5.88 D) compared to PKP (4.86 ± 4.97 D) (*p* = 0.241). Flattening occurred in 53.3% of PKP cases and 41.7% of LKP cases (*p* = 0.547), while steepening was slightly more frequent in LKP (58.3%) than in PKP (46.7%). The torque effect magnitude was comparable between PKP (3.85 ± 3.13 D) and LKP (3.59 ± 2.59 D) (*p* = 0.818). Counterclockwise (CCW) rotation was observed in 71.4% of PKP cases and 75.0% of LKP cases (*p* = 0.998), while clockwise (CW) rotation occurred in 28.6% and 25.0% of cases, respectively. Additional indices, including the flattening index (FI), curvature index (CI), irregularity of surface (IOS), and coma aberration (COA), showed no significant differences between the two techniques. The FI was slightly higher in LKP (0.91 ± 0.56) than in PKP (0.75 ± 0.43) (*p* = 0.413), while CI followed a similar trend (LKP: 0.74 ± 0.56, PKP: 0.45 ± 0.45, *p* = 0.157). IOS values remained close between groups (PKP: 0.72 ± 0.31, LKP: 0.76 ± 0.40, *p* = 0.794), and COA was slightly lower in LKP (2.30 ± 2.36) than in PKP (2.81 ± 4.56) (*p* = 0.735). Overall, both PKP and LKP demonstrated comparable outcomes in terms of visual performance, vector analysis, and optical indices, with no statistically significant advantage of one technique over the other.

## 4. Discussion

Arcuate keratotomy (AK) has long been used to treat astigmatism. While it is effective in reducing astigmatism, its predictability remains lower than that of other modern refractive techniques [1,2]. This study evaluated the outcomes of femtosecond laser-assisted arcuate keratotomy (AK), which has long been used to treat astigmatism. While it is effective in reducing astigmatism, its predictability remains lower than that of other modern refractive techniques [1,2,3]. This study evaluated the outcomes of femtosecond laser-assisted arcuate keratotomy (FSAK) in patients with post-keratoplasty astigmatism, demonstrating that FSAK can significantly improve uncorrected visual acuity (UCVA), reduce manifest astigmatism, and reshape corneal topography [2,4,5,6]. Our findings showed a statistically significant improvement in UCVA for the overall cohort, with the PKP subgroup achieving significance, while the LKP subgroup showed a favorable trend. Although best-spectacle-corrected visual acuity (BSCVA) did not improve significantly, the observed trend toward improvement likely reflects residual, undercorrected regular astigmatism. Reduction in the manifest cylinder was statistically significant overall, though not within individual subgroups.

Posterior corneal astigmatism remained largely unchanged, suggesting that the impact of FSAK is primarily limited to the anterior corneal surface [8]. LKP cases demonstrated greater surgically induced astigmatism (SIA), a higher rate of overcorrection, and a more pronounced myopic shift compared to PKP, which exhibited undercorrection [14,15]. This may reflect differences in biomechanical response based on graft architecture. Despite these differences, vector and efficacy indices were comparable between graft types. LKP showed slightly higher efficacy but reduced precision, as reflected by a higher index of success (IOS). Chang et al. reviewed published data and noted that near full-thickness FSAK incisions (up to 90%) provided greater correction (35.4% to 84.77% post-keratoplasty) but were associated with higher risks such as wound gape, infection, and overcorrection (19.4% to 43.5%) [12]. Intrastromal FSAK (IFSAK), which spares Bowman’s layer, avoids epithelial disruption and offers better safety but yields slightly less correction (23.53–89.42% post-keratoplasty) [12]. Our findings align with the literature in demonstrating differential responses between PKP and LKP, highlighting the importance of graft type in outcome variability [13,15,16].

The significant myopic shift observed postoperatively, particularly in the LKP group, is likely multifactorial. Corneal steepening following arcuate incisions, as evidenced by increased Kmax and Flat K values, is a key mechanism. In LKP, the preserved endothelium and stromal interface may facilitate more dynamic remodeling, which might contribute to a larger refractive shift [19,20]. Clinically, this has implications for preoperative planning, especially in patients aiming for spectacle independence. The differing responses to FSAK between PKP and LKP can be attributed to several structural and biomechanical factors. PKP replaces the entire cornea, leading to unpredictable healing across full-thickness grafts, while LKP preserves the host endothelium and posterior cornea, resulting in more stable biomechanical behavior [20,21]. Flattening of the steep meridian through arcuate incisions may be accompanied by steepening in the orthogonal meridian (coupling effect), which may be more pronounced in LKP due to greater posterior elasticity [21]. PKP tends toward undercorrection due to scar rigidity and biomechanical damping, whereas LKP tends to overcorrect due to increased corneal responsiveness [22,23]. LKP showed slightly better efficacy despite greater overcorrection, possibly due to better stromal regularity and optical quality. PKP outcomes may be limited by posterior irregularity and interface misalignment.

The clinical implications of our findings are particularly relevant for surgeons managing challenging refractive outcomes after keratoplasty. First, our data suggest that FSAK can be a viable first-line surgical intervention for moderate to high post-keratoplasty astigmatism, particularly in patients with regular topographic patterns and good contact lens-corrected visual potential. The significant improvement in UCVA with minimal complications supports the procedure’s utility in reducing dependence on corrective lenses, especially in contact lens-intolerant individuals [2,6,7,11].

Second, the differential responses between PKP and LKP should inform preoperative counseling and nomogram customization. In LKP, the tendency toward overcorrection and greater myopic shift suggests the need for more conservative arc lengths or shallower incision depths, whereas PKP eyes may require more aggressive treatment due to their biomechanical rigidity and tendency toward undercorrection [14,15,23]. Incorporating graft-specific modifications could enhance predictability and minimize postoperative refractive surprises [16,17]. Furthermore, the limited effect of FSAK on posterior corneal astigmatism emphasizes the importance of targeting the anterior corneal surface in treatment planning [8,21]. This may justify using anterior segment OCT or tomographic vector planning tools to refine nomogram-based approaches [19].

Emerging technologies such as artificial intelligence-driven nomogram refinement or intraoperative aberrometry could further enhance accuracy and outcomes [24]. Future studies should explore the long-term stability of FSAK, especially in younger keratoconus patients, and assess the role of combination procedures, such as FSAK with excimer-based treatments or phakic IOLs, to address residual ametropia [4,10]. Multi-center trials or prospective cohorts would provide higher levels of evidence to support standardized treatment protocols [12].

One case of infectious keratitis following FSAK was observed in our cohort, previously published by the authors [18], reinforcing the importance of maintaining strict aseptic technique and postoperative surveillance. Overall, the complication rate was low and consistent with previously published reports [10,12,13]. However, as with any incisional procedure, the risk of wound gape, regression, or overcorrection remains and must be weighed against the refractive goals. This study also has several limitations. The retrospective design and relatively small sample size may limit generalizability. In addition, the lack of standardized postoperative refractive targets and the absence of a unified nomogram may have contributed to variability in outcomes. Differences in surgeon experience, graft centration, and incision alignment could also affect vector parameters. Future prospective studies with nomogram stratification by graft type and topographic profile are warranted to refine planning and improve predictability.

## 5. Conclusions

This study reinforces the value of FSAK as an effective option for managing post-keratoplasty astigmatism. Both PKP and LKP patients showed improvements in uncorrected visual acuity, though only the PKP group reached statistical significance. LKP eyes demonstrated greater refractive shifts and a higher likelihood of overcorrection, reflecting enhanced biomechanical responsiveness. These findings underscore the importance of individualized planning based on graft type to optimize surgical outcomes and minimize refractive surprises. Further research incorporating long-term outcomes and advanced planning tools is warranted.

## Figures and Tables

**Table 1:** Visual acuity and refractive outcomes.

Characteristic	Pre-op Mean ± SD [Range]	Post-op Mean ± SD [Range]	*p*-Value (Paired Samples *t*-test)
UCVA LogMAR, Snellen equivalent	0.92 ± 0.33 [0.48–2.00] 20/165	0.58 ± 0.39 [0.00–1.30] 20/76	<0.001 *
BSCVA LogMAR, Snellen equivalent	0.32 ± 0.21 [0.00–0.88] 20/42	0.26 ± 0.22 [0.00–0.70] 20/36	0.158
M cylinder	−6.15 ± 2.75 [−12–−1]	−4.49 ± 2.92 [−12–−0.25]	0.037 *
Cylinder axis	96.48 ± 54.8 [5–180]	75.76 ± 64.25 [10–180]	0.524
Manifest sphere	−1.13 ± 3.3 [−10–5]	−2.72 ± 4.05 [−11–1.75]	0.040 *

UCVA: uncorrected visual acuity, BSCVA: best spectacle-corrected visual acuity, M: manifest. *: statistically significant *p*-value.

**Table 2:** Pre and postoperative tomographic features of all keratoplasty.

Tomographic Elements	Pre-Op	Post-op 1 Month	*p*	Post-Op 6 m	*p*	Last Visit 12 m ± 36	*p*
K max	48.31 ± 5.75	51.19 ± 7.96	0.003	50.88 ± 5.85	<0.001	51.7 ± 6.44	0.001
Flat K	41.21 ± 3.79	44.39 ± 4.61	<0.001	45.13 ± 4.09	<0.001	44.22 ± 2.78	0.001
Steep K	50.36 ± 3.03	49.09 ± 4.21	0.008	49.92 ± 4.05	0.008	48.72 ± 3.173	0.007
Anterior corneal cylinder	9.14 ± 3.1	5.05 ± 3.11	<0.001	4.83 ± 2.42	<0.001	4.86 ± 2.22	<0.001
Anterior steep axis	114.9 ± 47.01	123.7 ± 38.8	0.422	114.12 ± 51.4	0.954	117.93 ± 47.15	0.745
Posterior corneal astigmatism	1.4 ± 0.53	1 ± 0.46	0.133	0.89 ± 0.44	0.116	0.89 ± 0.33	0.118

K max: maximum keratometry, K: keratometry.

**Table 3:** Pre and postoperative tomographic features of postpenetrating keratoplasty.

Tomographic Elements	Pre-Op	Post-Op 1 Month	*p*	Post-Op 6 m	*p*	Last Visit	*p*
K max	50.38 ± 6.58	53.19 ± 9.41	0.02	52.15 ± 6.5	<0.004	53.59 ± 6.81	0.002
Flat K	40.98 ± 4.03	42.88 ± 4.58	<0.002	43.78 ± 3.59	<0.001	42.89 ± 2.52	0.007
Steep K	50.16 ± 2.61	50.93 ± 4.52	0.001	48.14 ± 2.24	0.01	46.85 ± 3.07	0.007
Anterior corneal cylinder	9.16 ± 3.26	5.68 ± 3.31	<0.001	4.43 ± 2.09	<0.001	4.54 ± 2.8	<0.001
Anterior steep axis	112.39 ± 44.79	118.08 ± 46.79	0.422	98.93 ± 64.8	0.954	108.81 ± 59.08	0.745
Posterior corneal astigmatism	1.48 ± 0.52	1.06 ± 0.47	0.003	0.7 ± 0.44	>0.05	0.87 ± 0.37	>0.05

K max: maximum keratometry, K: keratometry.

**Table 4:** Pre and postoperative tomographic features of post-lamellar keratoplasty.

Tomographic Elements	Pre-Op	Post-Op 1 Month	*p*	Post-Op 6 m	*p*	Last Visit	*p*
K max	45.8 ± 3.36	49.61 ± 5.9	0.003	49.72 ± 5.16	<0.001	49.52 ± 5.43	0.001
Flat K	41.59 ± 3.77	46.23 ± 4.25	<0.001	46.37 ± 4.26	<0.001	45.65 ± 2.37	0.005
Steep K	51.1 ± 3.23	50.93 ± 4.52	>0.05	51.55 ± 4.71	>0.05	50.87 ± 1.52	>0.05
Anterior corneal cylinder	9.53 ± 2.76	4.69 ± 2.82	<0.001	5.18 ± 2.71	<0.001	5.22 ± 1.3	<0.001
Anterior steep axis	119.17 ± 53.38	130.23 ± 30.48	0.422	128.15 ± 31.48	0.954	128.46 ± 26.57	0.745
Posterior corneal astigmatism	1.35 ± 0.53	0.98 ± 0.47	0.02	1.06 ± 0.37	0.01	0.92 ± 0.3	0.006

K max: maximum keratometry, K: keratometry.

**Table 5:** Success vs. non-success groups.

Characteristic	Success (72%) Mean ± SD	Non-Success (28%) Mean ± SD	Odds Ratio [95% CI]	*p*-Value
Age (years)	34.86 ± 7.52	39.00 ± 7.91	0.93 [0.83–1.04]	0.187
Gender, male %	70.6	29.4	1.07 [0.22–5.15]	0.936
Surgery, LKP %	53.8	46.2	0.26 [0.05–1.42]	0.122
UCVA LogMAR	0.97 ± 0.34	0.81 ± 0.31	5.99 [0.29–125.52]	0.249
BSCVA LogMAR	0.32 ± 0.22	0.32 ± 0.2	1.07 [0.02–47.61]	0.974
M cylinder	−6.39 ± 2.41	−5.56 ± 3.56	0.89 [0.65–1.22]	0.468
Cylinder axis	86.32 ± 56.76	120.63 ± 43.87	0.99 [0.97–1.00]	0.144
Manifest sphere	−0.62 ± 2.80	−2.34 ± 4.23	1.19 [0.90–1.57]	0.225
K max	48.72 ± 5.77	47.94 ± 6.17	1.03 [0.89–1.18]	0.738
Flat K	41.85 ± 3.48	40.44 ± 4.53	1.11 [0.88–1.40]	0.361
Steep K	50.01 ± 3.35	50.57 ± 2.93	0.94 [0.73–1.22]	0.657
Anterior corneal cylinder	8.15 ± 2.58	10.13 ± 3.95	0.80 [0.60–1.07]	0.130
Anterior steep axis	121.16 ± 48.57	102.22 ± 64.43	1.01 [0.99–1.02]	0.380
Posterior corneal astigmatism	1.35 ± 0.51	1.44 ± 0.65	0.73 [0.16–3.23]	0.674
Posterior steep axis	120.19 ± 48.82	98.82 ± 60.83	1.01 [0.99–1.02]	0.316

LKP: lamellar keratoplasty, UCVA: uncorrected visual acuity, M: manifest cylinder, K max: maximum keratometry, K: keratometry.

**Table 6:** Comparative outcomes between PKP and LKP.

Parameter	PKP Mean ± SD	LKP Mean ± SD	*p*-Value
Visual outcomes			
Safety index	0.96 ± 1.08	1.35 ± 1.2	0.415
Efficacy index	2.17 ± 2.22	3.00 ± 1.94	0.325
Vector analysis			
TIA (D)	8.84 ± 3.38	8.56 ± 2.80	0.822
Overcorrection, n (%)	4 (28.6)	6 (54.5)	0.241
Undercorrection, n (%)	10 (71.4)	5 (45.5)	
SIA Magnitude (D)	7.15 ± 4.59	8.65 ± 5.74	0.456
ME (D)	−2.26 ± 3.95	−0.15 ± 4.40	0.222
AE (°)	−4.33 ± 12.68	−13.84 ± 29.62	0.289
DV (D)	5.65 ± 2.40	6.00 ± 2.65	0.747
SIA Axis (°)	83.17 ± 53.27	64.93 ± 56.49	0.397
Flattening/Steepening Effect Magnitude (D)	4.86 ± 4.97	7.37 ± 5.88	0.241
Flattening, n (%)	8 (53.3)	5 (41.7)	0.547
Steepening, n (%)	7 (46.7)	7 (58.3)	
Torque Effect Magnitude (D)	3.85 ± 3.13	3.59 ± 2.59	0.818
CCW, n (%)	10 (71.4)	9 (75.0)	0.998
CW, n (%)	4 (28.6)	3 (25.0)	
FI	0.75 ± 0.43	0.91 ± 0.56	0.413
CI	0.45 ± 0.45	0.74 ± 0.56	0.157
IOS	0.72 ± 0.31	0.76 ± 0.40	0.794
COA	2.81 ± 4.56	2.30 ± 2.36	0.735

TIA: target-induced astigmatism, SIA: surgically induced astigmatism, ME: magnitude of error, AE: angle of error, DV: difference vector, FI: flattening index, CI: correction index, IOS: index of success, COA: correction angle, CCW: counterclockwise, CW: clockwise.

## Data Availability

The datasets generated during and/or analyzed during the current study are not publicly available due to legal issues and the policy of our institution.
